# The interclerkship ambulatory care track: attributes of a longitudinal integrated clerkship that inspire medical students to pursue careers in primary care and work with underserved

**DOI:** 10.15694/mep.2018.0000133.1

**Published:** 2018-06-15

**Authors:** John Yohan Rhee, Joe-Ann Moser, Phoebe Prioleau, Yasmin Meah, Allison Gault

**Affiliations:** 1 Icahn School of Medicine at Mount Sinai; 2 University Hospitals Rainbow Babies & Children’s Hospital

**Keywords:** longitudinal integrated clerkship, integrated curriculum, ambulatory medicine

## Abstract

This article was migrated. The article was marked as recommended.

Few longitudinal integrated clerkships (LICs) have increased the proportion of students who choose to go into primary care or work with underserved populations. A mixed-methods questionnaire was developed and sent to alumni (2006-2016) of the Interclerkship Ambulatory Care Tract (InterACT), a third-year clerkship in which students apply evidence-based medicine and chronic care model principles to outpatient longitudinal care. A likert scale was utilized for quantitative questions. Descriptive and thematic analyses were performed on the qualitative responses using a constant comparative approach. A majority (80%; 49/61) responded. Of the 44 physicians who responded to questions about current specialty, 75% indicated pediatrics, family medicine, or internal medicine. The majority of respondents (89%) reported that they care for patients considered to be medically underserved. Alumni overwhelmingly felt that the clerkship impacted the following: their specialty choice (71%, 34/48), and the population of patients they chose to take care of (80%, 39/49). The following attributes emerged from the qualitative questions as key determinants of future decisions regarding specialty and patient population: holistic patient care, strong mentorship, longitudinal patient relationships, and care of the homebound. These key attributes, if implemented in other LICs, may be a means to increase the number of medical students that choose to work in primary care fields and/or with underserved populations.

## Introduction

Various medical schools have implemented primary care tracks or programs with longitudinal care components. Longitudinal integrated clerkships (LICs) have grown immensely in the medical education world and are defined mainly by three key aspects: 1) medical students participate in the comprehensive care of patients over time; 2) medical students have continuing learning relationships with these patients’ clinicians, and 3) through these experiences, medical students meet the majority of the academic year’s core clinical competencies across multiple disciplines simultaneously ( Quinn et al., 2011). However, there is no article to date that has studied which core aspects of a longitudinal integrated clerkship (LIC) that increase interest in primary care and specifically working with underserved populations.

The Inter-Ambulatory Care Track Clerkship (InterACT) is a multi-disciplinary, ambulatory care, LIC for interested students during their third-year at the Icahn School of Medicine at Mount Sinai. The clerkship implements a longitudinal care curriculum that places third-year medical students at the center of care for community-dwelling patients with chronic illness; it substantially replaces the traditional inpatient model in an effort to improve the training of students committed to longitudinal primary care and provides unique opportunities for these medical students to learn and teach humanism, advocacy, and interdisciplinary care through facilitated group-learning exercises that they expressly navigate with the assistance of senior graduates of the program as well as the clerkship directors. Students partake in multiple aspects of the clerkship at the Mount Sinai Medical Center, a large urban tertiary medical center. Additionally, students engage in some community-based venues for family medicine and pediatrics.

Students are selected for the InterACT clerkship through a competitive application process. Approximately eight-to-twelve students are chosen each year by a committee that bases its decision on demonstrated interest in advocacy, community service, and the care of vulnerable persons with chronic illnesses.

The structure of the clerkship allows medical students to care for patients in several primary and specialty care practices; mentorship is provided by select attendings in different specialties and with varying clinic structures. These mentors are selected based on their commitment to the longitudinal aspect of the clerkship and their ability to foster independence in clinical management for participating students. The clerkship has ranged in length from eight-to-fourteen weeks in the eleven years of its existence, and these weeks are interspersed throughout the year between and within core clerkships that are required of all third-year students. Each week that students are on InterACT, students spend a half-day in the ambulatory practices in several of the following disciplines: general pediatrics, general medicine, family medicine, psychiatry, and surgery. In addition, students are required to spend a half-day each week in the home-based medical primary care program, the Visiting Doctors Program, and up to eight or more sessions in the school’s student-run free clinic, the East Harlem Health Outreach Partnership (EHHOP). Students carry a minimum of five longitudinal patients for the duration of their third year; a significant proportion of students will carry several more.

A unique aspect of the program that has developed over the years is the didactic curriculum, which happens on a half-day each week during clerkship. Students select topics grounded in core primary care topics identified as high-yield by the previous year’s InterACT cohort. Innovative teaching modalities, such as whiteboard talks or interactive activities are strongly encouraged and mentored in execution by InterACT alumni mentors. These sessions include self-critique as well as thorough feedback from the preceptors, classmates, and InterACT alumni mentors. The didactic component of this clerkship aims to prepare students to be facile educators in the classroom and clinical settings.

The purpose of this study was to survey all previous students who have participated in InterACT to distinguish what aspects of the program support its mission of encouraging more medical students to pursue primary care specialties and work with underserved populations. Of note, the specialties included as “primary care” in the literature are varied ( Jeffe, Whelan, & Andriole, 2010); for the purposes of this paper, we have included pediatrics, family medicine, internal medicine, and internal medicine/pediatrics as “primary care specialties”, but have also presented data from psychiatry, obstetrics and gynecology, and emergency medicine.

## Methods

The proposed research study is a retrospective cohort analysis of past medical students who took part in InterACT from 2006 to 2016 at a large, hybrid, urban medical center in New York City. A mixed-methods questionnaire was developed and distributed via SurveyMonkey to past InterACT participants. The initial goal of this survey was twofold: 1. identify changes in perspectives in primary care before and after the clerkship and 2. determine how the clerkship impacted choice in specialty. The survey was developed based on themes identified in the by reviewing the literature ( Ackermann & Comeau, 1996; Berman et al., 2012; Colgan et al., 2011; Julian, Riegels, & Baron, 2011; Kost et al., 2014; Quinn et al., 2011) and based on the mission of the InterACT program. The survey was reviewed by the co-authors multiple times before administration, and at least two of the co-authors were former InterACT students.

The survey included general demographics and multiple-choice questions regarding specialty choice, which were analyzed using descriptive statistics. Additionally, there were three open-ended questions which allowed for gathering of qualitative information on choice of specialty, decisions to work with underserved populations, and approach to medicine. Descriptive and thematic analyses were performed on the open-ended responses using a constant comparative approach ( Hewitt-Taylor, 2001) by the first author (JYR) by using open coding; the resultant themes were reviewed independently by a second team member (JM). The team (JYR, JM, AG) then met and discussed the codes; like codes were organized into categories and related within a coding framework. All transcripts were then analyzed using NVivo 11. All three questions were analyzed using the same codes as many of the same themes emerged across the three questions.

All information collected had no personal identifiers, and the data were maintained on a password-protected Excel file. If there was only one student in a particular year who entered a given specialty, data was compiled to include all years to ensure confidentiality in the final report. This study was granted Exempt status by the Mount Sinai Institutional Review Board (IRB-16-01033).

## Results

A majority (80%; 49/61) of alumni responded: 10% were medical students, 71% were residents/fellows, and 18% were attending physicians. When asked about their InterACT sites, 98% (48/49) had at least one session per week at a pediatric practice and 94% (46/49) had at least one session per week at the home-based primary care program.

**Figure 1.**
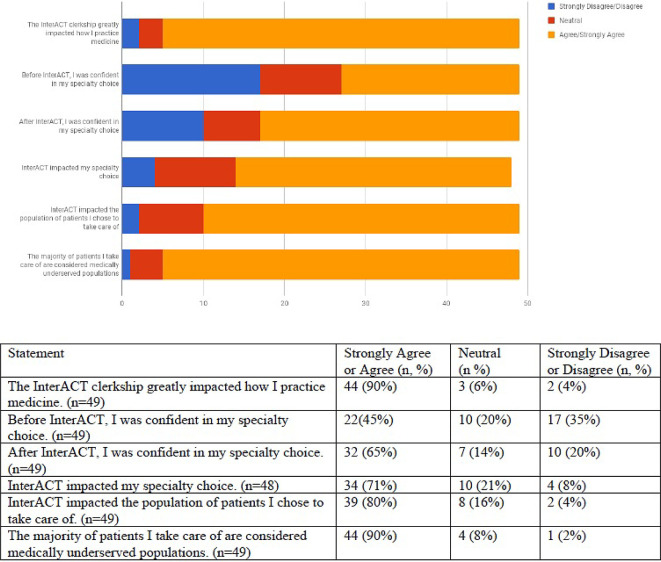
Rating of Statements on how InterACT impacted various career factors for participants on a Likert scale (Strongly Agree, Agree, Neutral, Disagree, Strongly Disagree)

Of the 44 physicians who responded to questions about current specialty, 75% indicated pediatrics, family medicine, or internal medicine; with an additional 18% indicated obstetrics and gynecology, psychiatry, or emergency medicine. Out of the 10 participants who indicated uncertainty in specialty at the time of entering InterACT, 60% responded that they eventually chose a primary care specialty. The majority of survey respondents (90%, 44/49) agreed or strongly agreed that they cared for patients considered to be medically underserved. A vast majority felt that the clerkship impacted: how they practice medicine (90%, 44/49), their specialty choice (71%, 34/48), and the socioeconomic population of patients they chose to take care of (80%, 39/49). (See Figure 1.)

### Qualitative Analysis: Impact of InterACT

The major qualitative themes of the impact of InterACT included the clerkship’s role in confirming participants’ decision to go into primary care and influencing their desire to care for the underserved.

*Primary care.* Approximately 20 respondents focused on how InterACT confirmed or increased their interest in primary care. They attributed this to the varied clinical site experiences within the clerkship; some gave examples of seeing children developing at their pediatric site, while others mentioned advocating for patients longitudinally at their family medicine or internal medicine clinics.

“ *[..] made me believe that primary care was a viable option. Showed me people who practiced primary care with joy.*

*Caring for the underserved.* A large majority of participants indicated that the clerkship reinforced their decision to care for the underserved; some even chose residencies with a focus on advocacy due to the clerkship.

*“The clerkship provided me with ample time with practitioners who worked at various sites in East Harlem. I was placed at [the hospital-based primary medical clinic] and a school based health center for medicine and [pediatrics]. The former gave me exposure to practice in a busy practice where physicians cared for a predominantly Medicaid population, and the latter was a small East Harlem elementary school---both revealed how poverty, the built environment, and deliberate limited access to healthcare, much of which was mediated through racism, had devastating health impacts on patients, their families, and an entire community.”*

### Qualitative Analysis: Attributes of a Successful LIC

Particularly influential aspects of the program included mentorship, learning about the holistic care of the person, the care of patients through the home-based primary care program, and the longitudinal provider-patient relationships.

*Impact of Strong Mentorship.* Mentorship was highlighted by participants, many of whom indicated that mentors not only played a role in developing stronger clinical skills, but also taught them how to provide high-quality patient-centered care. Mentors also crucially helped participants decide on their specialties and inspired them to work with the underserved.

*“InterACT provided me with role models unlike the other clerkships during 3rd year. The relationships I formed with my mentors helped me recognize the value in taking time thinking about, forming relationships, and making medical decisions regarding patients.”*

*“I always knew I wanted to work with underserved populations. InterACT helped me find mentors who were also dedicated to this as well as intensive experience with patients that reaffirmed the need for doctors who advocate for patients.”*

*Holistic care of the person.* Participants iterated how the program helped them focus on the whole patient. Specifically, they mentioned learning how socioeconomic factors can play a role in the patient’s health, and how to view patients in the context of their social determinants of health.

*“I now emphasize patient-centered metrics and have an ever-growing appreciation for the degree to which environment (physical or otherwise) has a profound impact on how, and when, patients interact with the health care system.”*

*Home-based Primary Care - A Unique Aspect of the InterACT LIC.* The Mount Sinai Visiting Doctors Program is a unique care model in which physicians visit homebound patients, providing comprehensive medical treatment for them outside of the hospital setting. Alumni resoundingly emphasized how involvement in the program had a profound impact on their current approach to medical care, and also their desire to work with the underserved.

*“Working at my Visiting Doctors site, a site where we performed the medical management for patients with significant mental illness, strengthened my desire to treat patients and families whose chronic medical concerns may be coupled with significant developmental delay or psychiatric illness secondary to the trauma of childhood illness.”*

*“The memory of my Visiting Doctors patient continues to serve as motivation for working with disadvantaged, and often marginalized, populations.”*

*The Longitudinal Care Relationship.* Participants frequently mentioned the emotional impact of developing longitudinal relationships with patients over the third-year clerkship, emphasizing its critical role in their formation as young doctors.

*“The opportunity to follow patients over time at this early stage in my training instilled a deep appreciation for the longitudinal doctor-patient relationship.”*

*“The experience of having continuity with primary care patients is uncommon as a medical student and one of the things that made InterACT so special. I still think about some of the patients I cared for--they were probably among the more formative clinical experiences I have had.”*

Other important themes that were highlighted included the importance of learning how to teach with evidence-based medicine and the unique clinical skills obtained.

*“InterACT gave me the tools for how to teach medicine and the importance of teaching in my career. It also showed me how to approach primary care with an evidence-based approach.”*

“ *InterACT encouraged me to reason and develop plans independently early on in my third year, and made me better equipped to provide both inpatient and outpatient care as a resident. My approach to follow up and inter-visit care is probably more hands on than it would otherwise have been because I had the opportunity to provide wrap-around care with my limited InterACT patient panel.*

## Discussion

Though other longitudinal care clerkships have shown improvements in professional performance and satisfaction, few have demonstrated an increase in the proportion of students deciding to go into primary care or work with underserved populations. ( Bell, Krupat, Fazio, Roberts, & Schwartzstein, 2008) Our survey showed that nearly three-fourths of students felt that the clerkship impacted their specialty choice, and an even greater percentage noted its influence in deciding to work for the underserved. Results from the qualitative analysis mirrored these findings, with the predominant themes being that InterACT confirmed participants’ interest in going into primary care and working with underserved patients.

**Figure 2.**
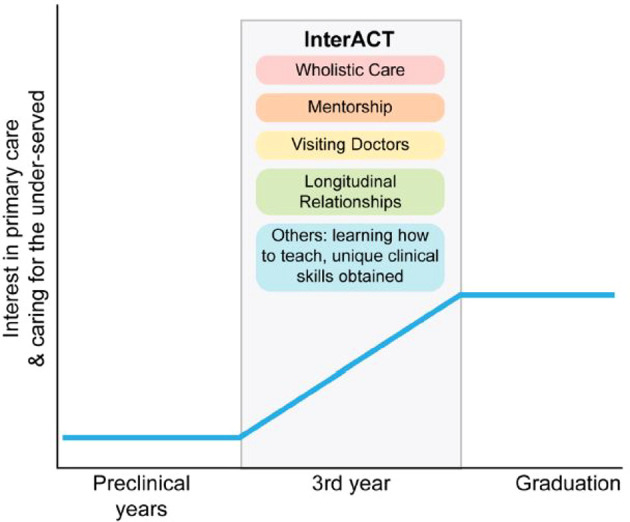
Themes that emerged from qualitative analysis of the open-ended questions: Key attributes of a successful LIC)

Although our original objective was not to determine key attributes of a successful LIC, themes that elucidated such components of InterACT emerged from qualitative analysis of the open-ended questions that were originally designed to measure the program’s impact on participants’ future career choices. These themes, such as longitudinal patient relationships, strong mentorship from attending physicians, and visiting patients in their homes, may serve as a framework for replicating similar clerkships at other medical schools across the country ( Figure 2). Support for the creation of such programs is especially important in the setting of a predicted severe shortage of primary care providers, with one article estimating that primary care residency production must increase by 21% compared to current production to meet the shortage by 2035 ( Petterson, Liaw, Tran, & Bazemore, 2015), and another showing an overall decline in generalist-primary care specialty choices since 1997 ( Jeffe et al., 2010).

Our findings corroborate previous research on other programs that have been designed to increase the number of medical students going into primary care. A program in California focused not only on creating a track for students to gain experience in primary care, but also included aspects of scholarly and leadership activities and focused mentorship( Eidson-Ton et al., 2016), and another program based out of Washington similarly engages students in scholarly output as well as a longitudinal component that spans throughout all four years of medical school ( Greer et al., 2016). Our study has shown, qualitatively and through the survey, that participants were more likely to choose primary care specialties and the longitudinal care component was an important driver of that interest.

There were a few limitations to our study. First, many of the students who chose to participate in InterACT may have done so with an already determined interest in primary care and working with underserved populations. The qualitative data supports this theory, but interestingly also demonstrates that many respondents thought that InterACT helped confirm their initial interest by allowing them to see themselves working as future primary care physicians. They indicated this was a result of their mentor interactions, as well as the longitudinal patient relationships, both of which are often missing in traditional medical school clerkships. Furthermore, the data showed that InterACT convinced a smaller group of students, who did not pursue primary care, the value of bringing a primary care focus to their chosen specialties, and out of a smaller subset of students with “undetermined” specialty choice at the start of InterACT, a majority decided afterwards to enter a primary care specialty. In addition, students also apply to InterACT with the intention of forming close relationships with clinical mentors, which is often difficult in the third-year medical school setting, and therefore, those who apply are not always solely interested in primary care.

Lastly, although preselection may present a bias the LIC creates a space in which to obtain a guided and in-depth experience in caring for vulnerable persons. The care of such persons is extremely challenging and we believe that just because a student, by virtue of being a trainee, may be exposed to such a population, the commitment to caring for such persons needs critical mentoring during medical school to stick. Many students may come in with the ideal to care for such patients in their careers but when circumstances change, that commitment can wane. An LIC that specifically focuses on this gives our students not only a supportive venue but also shapes and cements their commitment to primary care and caring for vulnerable persons.

Another limitation is that respondents were in different stages of training at the time the survey was administered. In order to ensure adequate response numbers, this was inherent to the study, and therefore not avoidable. We believe, however, that the diversity of perspectives provided by respondents at different levels of training added to the richness of the qualitative data.

Finally, the sample size was small. However, we still felt that the unique aspects of the InterACT curriculum are important to share in the literature, especially the Visiting Doctors Program and that this would contribute to the continued development of LICs in the medical education community. And though the sample was small, supplementing the qualitative to the quantitative questions provided a more robust view of the impact of InterACT.

Future steps include prospectively following the next InterACT cohort, to gather more in-depth information on career choice and how that changes over time prior to, during, and after completion of the InterACT clerkship as well as studying this model across multiple institutions. It would also be interesting to look at whether InterACT is associated with a lower level of medical student and resident burn-out, potentially due to the protective effect of creating and sustaining longitudinal patient relationships.

## Conclusion

An LIC with a commitment to mentor students to work with the underserved is one way to offer a venue that cements a more in-depth understanding of how to navigate and lead the care of such persons under close mentorship at a critical stage in training where students are developing a sense of professional identity and commitment. The aforementioned attributes are key components of a LIC that, if implemented in other LICs, may be a means to increase the number of medical students that choose to work in primary care fields and/or with underserved populations.

## Take Home Messages

• The aim of this study was to determine the key attributes of a longitudinal integrated clerkship (LIC), Interclerkship Ambulatory Care Tract (InterACT), that lead participants to pursue careers in primary care and work with underserved populations.
• InterACT, is a multidisciplinary, ambulatory care, third-year clerkship in which a select group of 8-12 students apply evidence-based medicine and chronic care model principles to outpatient longitudinal care.
• InterACT alumni overwhelmingly felt that the clerkship impacted the following: how they practice medicine (90%), their specialty choice (71%), and the population of patients they chose to take care of (80%).
• The following attributes emerged from the qualitative questions as key determinants of future decisions regarding specialty and patient population: holistic patient care, strong mentorship, longitudinal patient relationships, and care of the homebound through the Mount Sinai Visiting Doctors Program.

## Notes On Contributors

John Rhee, MD, received a BS in Policy Analysis and Management from Cornell University and was accepted into Mount Sinai’s Humanities and Medicine Early Acceptance Program and as a Dean’s Scholars in Global Health. He is currently a fourth-year medical student with an interest in neuro-palliative care.

Joe-Ann Moser is a medical student at the Icahn School of Medicine at Mount Sinai, Class of 2019. She participated in the Interclerkship Ambulatory Care Track (InterACT), a longitudinal clerkship focusing on ambulatory medicine. She is currently pursuing a scholarly year in the use of technology in medical education.

Phoebe Prioleau is an intern in the Department of Pediatrics at Rainbow Babies and Children’s Hospital in Cleveland, OH. She graduated from the Icahn School of Medicine at Mount Sinai with an MD/MPH. She is undecided on a specialty but is interested in pediatric nephrology.

Yasmin Meah is Associate Professor of Medicine, Co-Clerkship Director of the InterACT, and co-founder and Medical Director of the East Harlem Health Outreach Partnership. She received her BA from Johns Hopkins University, her MD from Harvard Medical School, and completed her residency in internal medicine at Mount Sinai.

Allison Gault is Assistant Professor of Pediatrics, Co-Clerkship Director of the InterACT, and Co-Director of Junior Urban Movement Program at Mount Sinai. Dr. Gault received her BA from Georgetown University and her MD from the Sackler School of Medicine at Tel Aviv University.
