# Supplementary material for: The interclerkship ambulatory care track: attributes of a longitudinal integrated clerkship that inspire medical students to pursue careers in primary care and work with underserved
Source: MedEdPublish (2016). 2018 Jun 15;7:133. [Version 1] doi: 10.15694/mep.2018.0000133.1 (PMC10699401; doi:10.15694/mep.2018.0000133.1)
Supplement: Peer review history

# Reviewer report: Reviewer response for version 1

**DOI:** 10.21956/mep.19567.r29026

**Published:** 2019-07-22

**Authors:** Trevor Gibbs

**Affiliations:** 1 AMEE

This review has been migrated. The reviewer awarded 4 stars out of 5 I enjoyed reading this paper and although there are some minor changes that could be made ( expanded on by my co-reviewers and which I do not wish to repeat) ) I did think that there was a very good message coming from this paper.To me, many LICs are designed but not always with purpose, based on faculty wanting to add an LIC to a curriculum. I think that this paper clearly shows that a clear design for purpose and having relevant learning outcomes within its structure, shows that LICs can be fit for purpose.I would encourage all those involved in curriculum design to read this paper- thank you

Reviewer Expertise:

NA

No decision status is available

# Reviewer report: Reviewer response for version 1

**DOI:** 10.21956/mep.19567.r29025

**Published:** 2019-03-08

**Authors:** Ken Masters

**Affiliations:** 1 Sultan Qaboos University

This review has been migrated. The reviewer awarded 3 stars out of 5 The paper is mostly well written on an important topic, examining core aspects of a longitudinal integrated clerkship (LIC) and its relationship to interest in primary care and working with underserved populations. Although the literature raised in the paper is rather sparse, the authors have clearly laid out the problem and the context in which they work.Some issues are:• The description of the Method should follow the chronological order of events as far as possible: in this case, the design of the instrument, and then its distribution, not the other way.• The statistics are presented far too broadly. For example, “75% indicated pediatrics, family medicine, or internal medicine” should be broken down; the other specialities should be similarly broken down, preferably in tabular form.• There should also be statistical tests performed on the impacts and relationship to specialty (Figure 1 should be expanded to indicate correlations, p values, etc).• Figure 2 should be placed into the Results.So, this is a useful paper, but it does need some tidying.

Reviewer Expertise:

NA

No decision status is available

# Reviewer report: Reviewer response for version 1

**DOI:** 10.21956/mep.19567.r29024

**Published:** 2018-06-17

**Authors:** Klaus von Pressentin

**Affiliations:** 1 Stellenbosch University

This review has been migrated. The reviewer awarded 3 stars out of 5 Thank you for presenting the findings from your programme evaluation. It would have been helpful to know how many students were part of the retrospective cohort who have participated in the InterAct clerkship. The recruitment process is unclear: it was mentioned that it was distributed via SurveyMonkey to past students, but which proportion participated? The numbers selected to the programme were 8 – 12 students per year for the 11 years analysed (2006 – 2016). This meant that the population were at least 88 students, of which 61 participated (a response rate of 69%). The response rate helps one to interpret how representative the quantitative survey data is. It would have been interesting to know why past students elected not to participate, especially as the numbers are so small. The InterAct programme seems to have a strict selection process, and the limitation of selection bias was mentioned. How many students were in a typical year group from which the selection was made? Again, more information on the total population would have been helpful.The InterAct programme is making a difference in the InterAct participants’ career choice. The qualitative findings are useful and the researchers have mentioned potential ideas for future research. It will be useful to also conduct interviews with InterAct faculty/mentors, as well as future employers of the graduates (and possibly patients and community representatives).Finally, the diversity of the research team members is a positive finding, as the inclusion of past and present InterAct participants shows a commitment to involving graduates in the community of practice. It also helps with understanding the reflexivity of the authors from the readers’ perspective.It would be interesting to see if the InterAct programme will be able to accommodate a larger proportion (if not the 100%) of each year group, as one would like to see that exposure to primary care and working with underserved populations becomes the standard during the medical curriculum.

Reviewer Expertise:

NA

No decision status is available
